# Quantitative circumferential strain analysis using ATP-stress/rest 3-Tesla tagged magnetic resonance to evaluate regional contractile dysfunction in ischemic heart disease

**DOI:** 10.1186/1532-429X-17-S1-Q64

**Published:** 2015-02-03

**Authors:** Masashi Nakamura, Tomoyuki Kido, Teruhito Mochizuki

**Affiliations:** 1 Radiology, Ehime University, Toon, Japan; 2 Saiseikai Matsuyama Hospital, Matsuyama, Japan

## Background

We evaluated whether quantitative circumferential strain (C-strain) analysis using adenosine triphosphate (ATP)-stress/rest 3-Tesla (3-T) tagged magnetic resonance (MR) can depict myocardial ischemia as contractile dysfunction on stress. We evaluated whether it can differentiate non-ischemia, myocardial ischemia and infarction. We assessed its diagnostic performance in comparison with ATP-stress myocardial perfusion MR and late gadolinium enhancement (LGE)-MR.

## Methods

In 38 patients suspected of having coronary artery disease (CAD), tagged MR and perfusion MR under ATP-stress and rest conditions and LGE-MR imaging were performed. The peak negative value (%) of the circumferential strain (C-strain) during a cardiac cycle and the time-to-peak C-strain were measured in the left ventricle using short-axis tagged images during ATP-stress and at-rest conditions. Myocardial segments were categorized as non-ischemic (n = 485), ischemic (n = 74), or infarcted (n = 49) from the results of perfusion MR and LGE-MR.

## Results

In non-ischemic segments, C-strain was significantly greater during ATP-stress (-15.9 ± 3.1%) (mean ± SD) than at-rest (-14.0 ± 3.2%, p < 0.001) imaging. Conversely, in ischemic segments, C-strain was significantly lower during ATP-stress (-13.9 ± 3.2%) than at-rest (-15.4 ± 3.1%, p < 0.01) imaging.

Under both ATP-stress and at-rest conditions, C-strain values in infarcted segments were significantly lower than those in non-ischemic and ischemic segments. Under ATP-stress, C-strain in non-ischemic segments was significantly greater than that in ischemic segments. However, under at-rest conditions, there was no significant difference between ischemic and non-ischemic segments.

Cutoff values of -12.0% for at-rest C-strain and 49.4% for at-rest time-to-peak C-strain allowed differentiation between infarcted segments from non-ischemic and ischemic segments with sensitivities of 79% and 61%, specificities of 76% and 91%, accuracies of 76% and 88%, and areas under the curve (AUCs) of 0.81 and 0.75, respectively. The differences in C-strain values between ATP-stress and at-rest conditions (stress−rest C-strain) in non-ischemic segments (−1.78 ± 2.45%) were significantly smaller than in segments with ischemia (+1.47 ± 1.89%, p < 0.001). A cutoff value of +0.3% for the stress−rest C-strain value could differentiate segments with ischemia from non-ischemic segments with a sensitivity of 75%, a specificity of 82%, an accuracy of 82%, and an AUC of 0.86.

## Conclusions

C-strain analysis using tagged MR can quantitatively assess contractile dysfunction in ischemic and infarcted myocardium.

## Funding

N/A.

**Figure 1:**
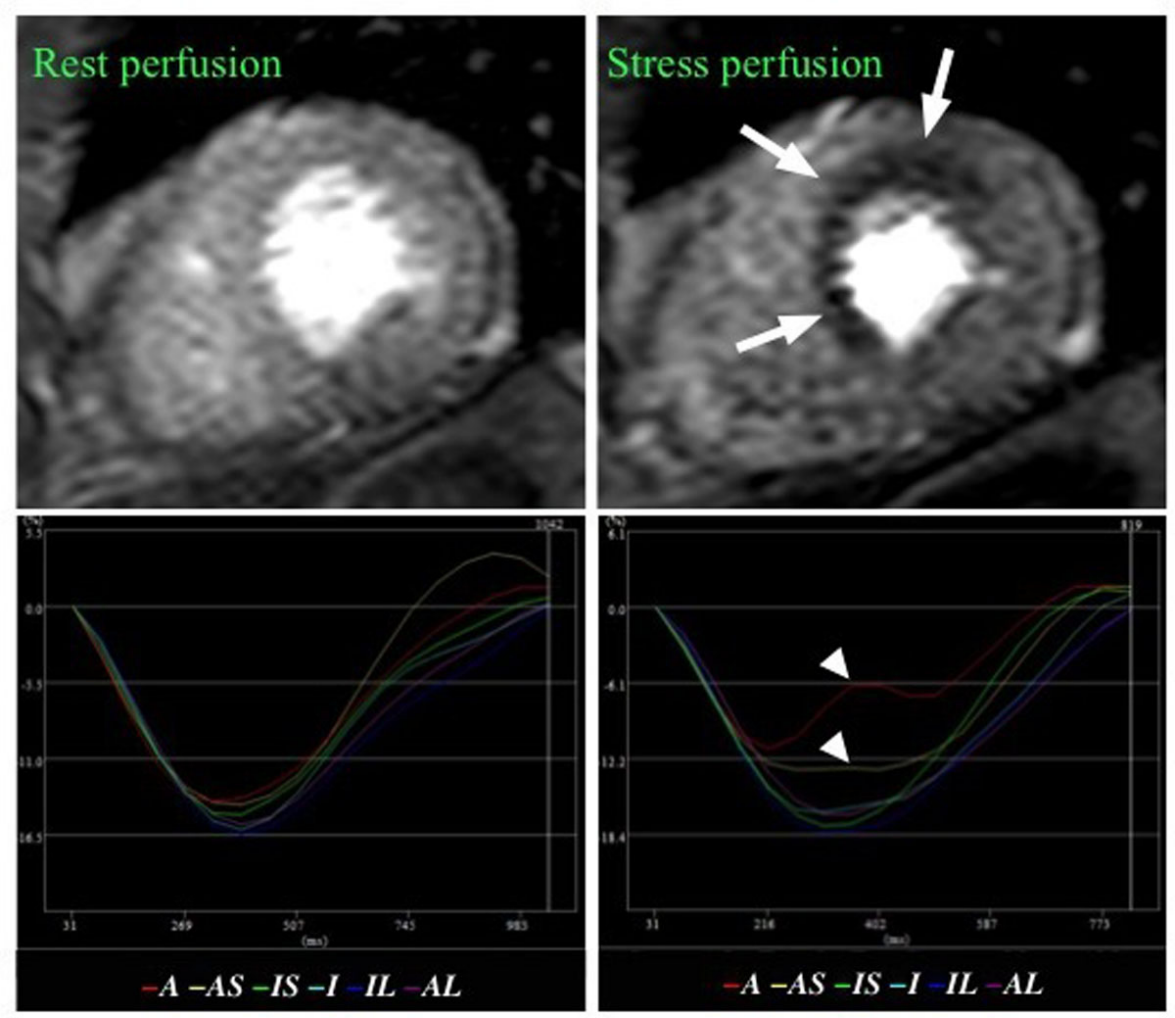
A, anterior; AS, anteroseptal; IS, inferoseptal; I, inferior; IL, inferolateral; AL, anterolateral

